# Incidental ureteral triplication discovered during surgery for severe hydronephrosis: a case report

**DOI:** 10.1097/RC9.0000000000000507

**Published:** 2026-05-07

**Authors:** Mohammad Shafiqi, Mujtaba Yama, Dunya Moghul

**Affiliations:** a Department of Pediatric Surgery, Afghan Arya Specialty Hospital, Herat, Afghanistan; b Independent Researcher

**Keywords:** case report, congenital anomaly, hydronephrosis, low-resource setting, ureteral triplication

## Abstract

**Introduction:**

Ureteral triplication is an exceptionally rare congenital anomaly of the upper urinary tract, with fewer than 100 cases reported worldwide. Preoperative diagnosis remains challenging, particularly in low-resource settings.

**Presentation of Case:**

A 3.5-year old boy presenting with dysuria and intermittent severe left flank pain. Initially diagnosed as ureteral duplication with hydronephrosis. Due to limited access to advanced imaging modalities, no further diagnostic evaluation was performed. Intraoperative findings revealed type 3 ureteral triplication with marked hydroureteronephrosis of the upper moiety and non-functioning moiety. The patient underwent a successful partial nephroureterectomy.

**Discussion:**

Ureteral triplication may be misdiagnosed as duplication, especially when advanced imaging is unavailable. In such settings, improved ultrasonographic assessment and increased clinical awareness are essential. Low-cost strategies, including standardized ultrasound protocols and tele-radiology consultation, may enhance diagnostic accuracy.

**Conclusion:**

This case shows that ureteral triplication can be missed when relying only on ultrasound, especially in resource-limited settings. Improved training and careful evaluation can help detect such anomalies earlier and guide better management.

## Introduction

Triplication of the ureter is a very rare upper urinary tract anomaly, with nearly 100 cases reported between 1870 and 2000^[^1^]^. According to Smith’s classification, this condition can be anatomically divided into four types: (1) complete triplication, with three ureters and three bladder orifices (35% of cases); (2) incomplete triplication, in which two of the three ureters join along their course to the bladder, resulting in two orifices (21%); (3) trifid ureters, which unite and drain through a single orifice (31%); and (4) double kidney ureters with one bifurcation forming an inverted Y draining into three orifices (9%)^[^2^]^.

Clinical presentation varies: while 8% of cases remain asymptomatic, most patients experience recurrent urinary tract infections, urinary incontinence, or renal colic^[^3^]^. Ureteral triplication is often associated with other urological anomalies, including contralateral duplication, ureteral ectopia, vesicoureteral reflux, and renal anomalies^[^1,4^]^.

Diagnosis requires thorough investigations, including laboratory studies, ultrasonography, nuclear renal scans, and contrast studies such as intravenous urography, CT or MR urography, and voiding cystourethrography. Urethrocystoscopy and retrograde pyelography are particularly valuable for delineating the exact configuration of ureteral triplication, especially in patients with impaired renal function^[^5^]^.

HIGHLIGHTS

- Ureteral triplication is an extremely rare congenital urinary tract anomaly.
- Most patients are symptomatic; only ~ 8% of cases remain asymptomatic.
- Preoperative diagnosis is challenging and often confused with ureteral duplication.
- Partial nephroureterectomy is a viable treatment for non-functioning moieties.
- Limited diagnostic resources in Afghanistan delayed accurate preoperative diagnosis.

Here, we report a case of type 3 ureteral triplication associated with severe hydronephrosis of the upper moiety.

This case report has been reported in line with the SCARE 2025 criteria^[^6^]^.

## Description of case

A 3.5-year-old boy presented with dysuria (manifested as irritability during urination) and intermittent episodes of severe pain, characterized by crying, restlessness, and guarding of the left flank over 7 weeks. There was no history of recurrent urinary tract infections, hematuria, or urinary incontinence. On physical examination, the patient was stable, with normal vital signs and no palpable abdominal masses. His weight was 14 kg, and his growth and developmental milestones were appropriate for age. The patient had no significant past medical or surgical history. He had no prior hospitalizations or chronic illnesses. He was not taking any regular medications and had no known drug allergies. The child belonged to a low socioeconomic background, with limited access to advanced healthcare facilities. There was no family history of congenital urinary tract anomalies or hereditary diseases. Laboratory Results: Urinalysis (UA) showed significant bacteriuria (++), with white blood cells (WBC) of 10–15 per high-power field. Kidney function tests revealed a serum creatinine level of 0.5 mg/dL and a blood urea nitrogen level of 13 mg/dL. Complete blood count (CBC) showed a hemoglobin (Hb) level of 11.6 g/dL, WBC of 8400/µL, and platelets of 340 000/µL.

Renal ultrasonography demonstrated a left duplex kidney with severe hydronephrosis of the upper moiety. Due to the limited availability of advanced imaging modalities such as CT urography or intravenous pyelography in our region, further radiological evaluation was not feasible. Surgical exploration was therefore undertaken based on the ultrasound findings.

**Figure 1.**
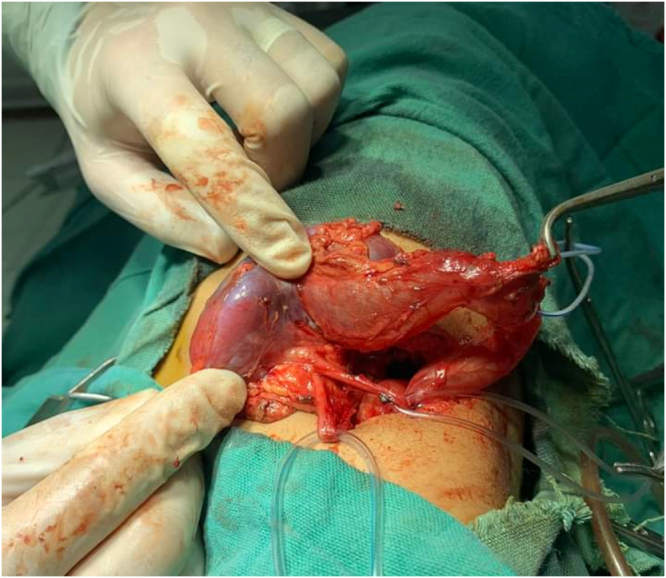
Intraoperative findings in our patient showing ureteral triplication. The upper pole moiety demonstrates marked hydroureteronephrosis , while the two additional ureters are identified separately, confirming the presence of three distinct ureteral channels.

**Figure 2.**
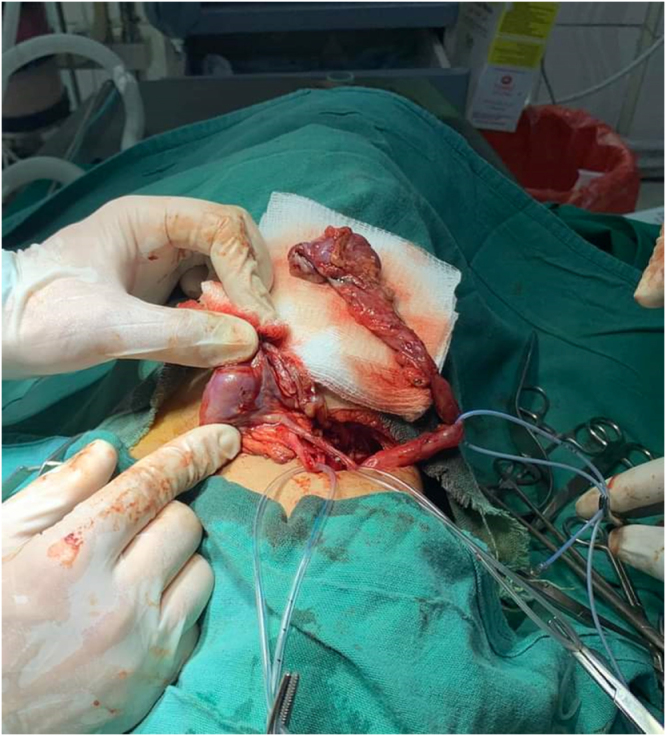
Intraoperative image demonstrating surgical excision of the severely hydronephrotic upper pole moiety as part of definitive management for the non-functioning segment.

**Figure 3.**
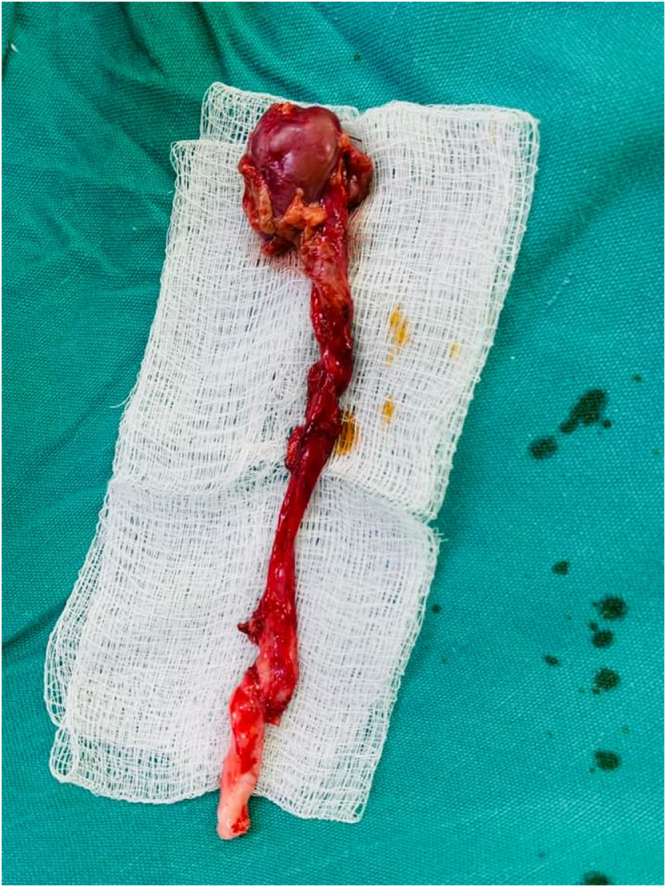
Intraoperative view after completion of the upper moiety partial nephroureterectomy. The diseased upper pole and its ureter have been excised, leaving the remaining moieties and ureters intact.

Intraoperatively, a triple collecting system was identified. The upper moiety showed severe hydronephrosis and hydroureter, with markedly thinned renal parenchyma. The other two ureters appeared normal. Partial nephroureterectomy was chosen due to the severely dilated upper moiety with markedly thinned renal parenchyma, indicating a non-functioning segment. The remaining functional renal tissue was preserved, so this approach was the most appropriate surgical option (Figure 1–3).

Postoperatively, the patient was managed with antibiotics and analgesics. He had an uneventful recovery and was discharged on the fifth postoperative day. At follow-up, he remains asymptomatic and under regular surveillance. Screening of family members with renal ultrasonography showed no abnormalities.

## Discussion

We report a rare case of ureteral triplication presenting with dysuria and severe left flank pain, managed by partial nephroureterectomy. This case is noteworthy because the anomaly was initially misdiagnosed as ureteral duplication with severe upper moiety hydronephrosis, and the true anatomy was only recognized intraoperatively, significantly influencing surgical decision-making.

Ureteral triplication is among the rarest anomalies of the upper urinary tract and is thought to arise from either multiple ureteric buds or early branching during embryogenesis^[^7^]^. Most patients are symptomatic, commonly presenting with recurrent urinary tract infections, incontinence, or flank pain, while only a small proportion remain asymptomatic^[^8^]^. Although multiple imaging modalities – including ultrasonography, intravenous urography, CT or MR urography, cystoscopy, and retrograde pyelography – can aid diagnosis^[^9^]^. Accurate preoperative identification remains challenging, particularly in resource-limited settings.

In the present case, the upper moiety was non-functioning, most likely due to chronic obstruction associated with severe hydronephrosis, which led to progressive parenchymal thinning and loss of renal function. This is consistent with previously reported cases where prolonged obstruction or ectopic drainage results in a poorly functioning renal segment^[^10^]^. Given the absence of functional renal tissue and the presence of persistent symptoms, partial nephroureterectomy was favored over reconstructive options. Reconstructive procedures such as ureteroureterostomy or ureteral reimplantation are typically considered when the affected moiety retains function; however, in this case, preservation was unlikely to provide clinical benefit and could increase the risk of recurrent infection or complications. Therefore, excision of the non-functioning segment represented the most appropriate and definitive management strategy^[^1^]^.

A major limitation in this case was the absence of advanced preoperative imaging such as CT urography or intravenous pyelography^[^11^]^, which contributed to the initial misdiagnosis. This reflects a broader challenge in low-resource settings, where diagnostic decisions often rely primarily on clinical evaluation and ultrasonography^[^12,13^]^.

In settings such as Afghanistan, improving preoperative detection requires practical and scalable strategies directly applicable to resource-constrained environments. First, standardized ultrasonography protocols should be implemented, ensuring routine and systematic evaluation of the renal collecting system and ureteral anatomy in all suspected urinary tract anomalies. Second, targeted training programs for radiologists and sonographers are essential to enhance recognition of complex anomalies such as duplex or triplex systems, particularly when advanced imaging is unavailable. Third, the adoption of tele-radiology consultation networks would allow peripheral centers to obtain expert interpretation of imaging studies, reducing diagnostic uncertainty. Finally, establishing structured referral pathways for suspected complex anomalies can facilitate timely transfer to higher-level centers before surgical intervention. These measures directly address the diagnostic gap observed in this case and may significantly reduce intraoperative surprises and improve patient outcomes.

Unlike most cases reported in the literature, where diagnosis is established preoperatively using advanced imaging, our case required intraoperative recognition. This highlights the critical importance of strengthening preoperative diagnostic pathways, particularly in low-resource settings, to enable accurate anatomical delineation and optimal surgical planning.

## Conclusion

This case demonstrates that ureteral triplication can be misdiagnosed when evaluation relies solely on ultrasonography in resource-limited settings such as Afghanistan. A key lesson is the need for systematic training of pediatric radiologists and sonographers to improve assessment of ureteral anatomy on ultrasound and to maintain suspicion when findings are atypical. Strengthening these low-cost diagnostic approaches can improve preoperative recognition of complex anomalies and support more appropriate management.

## Data Availability

The data supporting the findings of this study are available within the article. No additional data are available.

## References

[1] Al-Zubi M Al Faqieh A Altamimi O. Unilateral triplicate ureter with ipsilateral ureterocele a case report. Int J Surg Case Rep 2020;70:178–81. 32417734 10.1016/j.ijscr.2020.04.048 PMC7229424

[2] Singh TR Kumar DA Agarwala S. Triplication of ureter a rare case. J Indian Assoc Pediatr Surg 2022;1:91–93. 10.4103/jiaps.JIAPS_31_20 PMC8853593 35261520

[3] Wahyudi I Fahri M Situmorang GR. Bilateral ureteral triplication: a case report. Urol Case Rep 2020;33:101406. 33102104 10.1016/j.eucr.2020.101406 PMC7574039

[4] Akdogan N Gokalp F Izol V. Ureteral triplication associated with upper pole ureteropelvic junction stenosis. J Med Cases 2018;9:195–97.

[5] Ahmad Alfaseh AS. AI. A rare caseof triplicate ureter. Images Clin Urol 2018;121:1–2. 10.1016/j.urology.2018.09.003 30219557

[6] Kerwan A Al-Jabir A Mathew G. Revised surgical CAse REport (SCARE) guideline: an update for the age of Artificial intelligence. Prem J Sci 2025. doi:10.70389/pjs.100079

[7] Butorac D Djaković I Malčić J. Ureteral triplication accidentally found during cervical carcinoma operation. Acta Clin Croat 2021;60:329–31. 34744288 10.20471/acc.2021.60.02.24 PMC8564831

[8] Dumra A Dumra A Ramdev P. Persistent incontinence following surgery for ureteric triplication with contralateral duplication-A management dilemma. Urology 2019;134:221–24. 31494213 10.1016/j.urology.2019.08.040

[9] Liu S Lu R Guo Y. Laparoscopic triple-ureteral ureteroureterostomy in a patient with ureteral triplication: a case report. Medicine (Baltimore) 2022;101:e31580. 36343058 10.1097/MD.0000000000031580 PMC9646508

[10] Sion AE McClure C Campbell T. Type I ureteral triplication in an adult associated with an obstructed extravesicular megaureter surgically managed with partial nephrectomy. Cureus 2024;16:e51864. 38327927 10.7759/cureus.51864 PMC10848881

[11] Shafiqi M Yama M Rayan O. Delayed diagnosis of congenital cystic adenomatoid malformation as pneumonia: a case report. Int J Surg Case Rep 2025;128:111109. 40037270 10.1016/j.ijscr.2025.111109 PMC11925165

[12] Moghul D Hsu PJ Bryce E. Overcoming political upheaval to deliver pediatric surgical care in Afghanistan: prospective analysis of the first 1,000 procedures. J Am Coll Surg 2025;240:876–82. 39927655 10.1097/XCS.0000000000001350

[13] Moghul D. Mission impossible, made possible: two GAP fellows escape from Afghanistan. J Pediatr Surg 2022;57:1189–95. 35410710 10.1016/j.jpedsurg.2022.02.021

